# Enhanced extracellular production of laccase in *Coprinopsis cinerea* by silencing chitinase gene

**DOI:** 10.1007/s00253-024-13164-9

**Published:** 2024-05-07

**Authors:** Dongbang Yao, Yuting Ma, Jie Ran, Jiaxiu Wang, Ursula Kües, Juanjuan Liu, Danya Zhou, Xuecheng Zhang, Zemin Fang, Yazhong Xiao

**Affiliations:** 1 School of Life Sciences, Anhui University, Hefei, 230601 China; 2 Anhui Key Laboratory of Biocatalysis and Modern Biomanufacturing, Hefei, 230601 China; 3 AHU Green Industry Innovation Research Institute, Hefei, 230088 China; 4 Molecular Wood Biotechnology and Technical Mycology, Büsgen-Institute and Goettingen Center for Molecular Biosciences, University of Goettingen, Büsgenweg 2, 37077 Goettingen, Germany; 5 School of Basic Medical Sciences, Zhengzhou University, Zhengzhou, 450001 China

**Keywords:** Laccase, *Coprinopsis cinerea*, Chitinase, RNAi, Extracellular production, 3-L fermenter fermentation

## Abstract

**Abstract:**

Laccase, a copper-containing polyphenol oxidase, is an important green biocatalyst. In this study, Laccase Lcc5 was homologous recombinantly expressed in *Coprinopsis cinerea* and a novel strategy of silencing chitinase gene expression was used to enhance recombinant Lcc5 extracellular yield. Two critical chitinase genes, *ChiEn1* and *ChiE2*, were selected by analyzing the transcriptome data of *C. cinerea* FA2222, and their silent expression was performed by RNA interference (RNAi). It was found that silencing either *ChiEn1* or *ChiE2* reduced sporulation and growth rate, and increased cell wall sensitivity, but had no significant effect on mycelial branching. Among them, the extracellular laccase activity of the *ChiE2*-silenced engineered strain Cc*lcc5*-anti*ChiE2*-5 and the control Cc*lcc5*-13 reached the highest values (38.2 and 25.5 U/mL, respectively) at 250 and 150 rpm agitation speeds, corresponding to productivity of 0.35 and 0.19 U/mL·h, respectively, in a 3-L fermenter culture. Moreover, since Cc*lcc5*-anti*ChiE2*-5 could withstand greater shear forces, its extracellular laccase activity was 2.6-fold higher than that of Cc*lcc5*-13 when the agitation speed was all at 250 rpm. To our knowledge, this is the first report of enhanced recombinant laccase production in *C. cinerea* by silencing the chitinase gene. This study will pave the way for laccase industrial production and accelerate the development of a *C. cinerea* high-expression system.

**Key points:**

• *ChiEn1 and ChiE2 are critical chitinase genes in C. cinerea FA2222 genome.*

• *Chitinase gene silencing enhanced the tolerance of C. cinerea to shear forces.*

• *High homologous production of Lcc5 is achieved by fermentation in a 3-L fermenter.*

**Supplementary information:**

The online version contains supplementary material available at 10.1007/s00253-024-13164-9.

## Introduction

Laccase (EC 1.10.3.2) is a copper-containing polyphenol oxidase, which is considered a green catalyst with important industrial applications due to its ability to oxidize phenols, carboxylic acids, and aromatic amine compounds and their derivatives, etc., with water as the only by-product (Barber-Zucker et al. 2022; Cascelli et al. 2023). There are abundant sources of natural laccase, with more than 80% of the laccase discovered to date derived from fungi (Curran et al. 2022). Among them, basidiomycetes laccase occupies over 90% of the current laccase market share due to its wide substrate range, high specific activity, and high redox potential (Loi et al. 2021). However, the yield and efficiency of laccase naturally secreted by basidiomycetes are relatively low. For example, even after 6 days of culture in mKjalke medium at 25 °C, the highest extracellular laccase activity of *Coprinopsis cinerea* was only 3.5 U/mL (Rühl et al. 2018). This will lead to a high production cost of basidiomycetes laccase, thus severely limiting its industrial application. High expression production of basidiomycetes laccase is therefore important.

Heterologous recombinant expression of basidiomycetes laccase in ascomycetous yeasts and filamentous ascomycetes (e.g., *Aspergillus*) has usually been used for its efficient production (Li et al. 2022b; Savinova et al. 2020). However, compared to natural laccase, the enzymatic properties of the recombinant laccase may be altered in terms of protein molecular weight, redox potential, pH stability, and thermal stability due to irrational post-translational modifications such as incomplete folding and over-glycosylation (Xu et al. 2019b; Zhuo et al. 2018). This seriously reduces the practical application value of the recombinant laccase obtained. On the contrary, using basidiomycetes homologous recombinant expressed basidiomycetes laccase can effectively solve the above-mentioned problems.

*C. cinerea*, a model microorganism for studying basidiomycetes genetic development, is an important basidiomycete chassis host with advantages of high transformation efficiency and short growth cycle (Liu et al. 2022; Pareek et al. 2022). Hitherto, a series of laccase genes under the control of *Agaricus bisporus gpdII* promoter have been successfully expressed in *C. cinerea* (Dörnte and Kües 2016; Rühl et al. 2018). Moreover, numerous strategies have been developed to increase the recombinant expression level of basidiomycetes laccase in *C. cinerea*, which mainly includes optimization of the host (Zhang 2022), promoter (Kilaru et al. 2006), signal peptide (Schulze et al. 2019), and medium components and fermentation conditions (Rühl et al. 2018). Among them, expression host optimization is limited to selecting different natural microorganisms as chassis without engineering them.

Current strategies to improve recombinant protein expression based on filamentous fungal genetic engineering modifications include knocking out host proteases, modulating protein post-translational folding and secretion process, and modifying fungal morphology (Li et al. 2020; Qian et al. 2019; Wang et al. 2020). Among them, mycelial morphology affects the yield and productivity of target products synthesized by filamentous fungi by influencing heat, mass, and momentum transfer under submerged fermentation (Gong et al. 2023). For example, the protein concentration in *Aspergillus niger* extracellular culture supernatant increased twofold by promoting the formation of small mycelial pellets and hyperbranched phenotype (Cairns et al. 2019). Therefore, achieving optimal mycelial morphology during submerged fermentation is important to improve targeted protein yield.

Filamentous fungi mycelial growth and morphogenesis involve the remodeling of cell wall structures. Chitin is a major structural component of fungal cell walls, and its content is mainly regulated by the activities of chitin synthases (EC2.4.1.16) and chitinases (EC3.2.1.14). Among them, chitin synthases are a family of membrane-embedded enzymes that catalyze the synthesis of chitin at cell wall expansion sites (Gong et al. 2023). Fungal chitinases are members of the glycosyl hydrolase (GH) family 18 and are involved in the depolymerization of fungal cell wall chitin polymers (Jeong et al. 2023). The most widely accepted view of fungal cell wall expansion is that cell wall–loosening enzymes (e.g., chitinases and glucanases) are involved in breaking down polysaccharide chains (e.g., chitin and *β*-1,3-glucan) to allow incorporation of newly deposited material while generating free ends that act as substrates for cross-linking enzymes (e.g., chitin synthases) to rigidify the cell wall (Riquelme et al. 2018). Therefore, regulating the expression level of chitin synthase or chitinase would be an effective strategy to obtain high yields of the target product in basidiomycetes.

Previous studies have modulated filamentous fungal hyphae morphology from the chitin synthesis perspective by regulating chitin synthase activity or its transcription factors expression levels to increase the target product yield (Gong et al. 2023; Shu et al. 2022). For example, by deleting *A. niger* chitin synthase activator Chs3, a higher number of smooth pellets were obtained while increasing citric acid production by 39.25% (Jiang et al. 2022). On the contrary, increasing the target product expression based on the chitinase degradation of chitin has not yet attracted much attention, and previous studies on chitinase have been limited to the investigation of its physiological function in fungal stalk growth (Niu et al. 2017).

A previous study showed that the *C. cinerea* laccase Lcc5 has a strong ethanol tolerance (Kilaru 2006). In addition, we recently found that Lcc5 has a significant detoxification effect on aflatoxin (Data unpublished). The above results suggest that Lcc5 has an important application value in wine lees feed processing. This study aimed to achieve efficient homologous recombinant expression of Lcc5 in the *C. cinerea* by silencing the chitinase gene. To that end, we first excavated the chitinase gene with the highest transcriptional activity in the *C. cinerea* FA2222 genome and then silenced its expression through RNA interference (RNAi). Next, the effects of chitinase gene silencing on mycelial growth, morphology, and recombinant expression of Lcc5 were investigated. Finally, the obtained recombinant *C. cinerea* potential for Lcc5 production was validated in a 3-L fermenter.

## Materials and methods

### Strains and media

*Saccharomyces cerevisiae* Y1H and *Escherichia coli* JM109 were purchased from Takara Biological Co., Ltd (Dalian, China) and were used for the construction and preparation of recombinant vectors, respectively. *C. cinerea* FA2222 (*A5*, *B6*, *acu1*, *trp1-1,1–6*; DSM 28333) was used for recombinant expression of Lcc5. LB and PDA medium were used to culture *E. coli* JM109 and *S. cerevisiae* Y1H, respectively. YMG and mKjalke medium were used to culture *C. cinerea* (Rühl et al. 2018). VI medium plate (per liter, 6 g D(-)-aspartic acid, 0.2 mg vitamin B1, 4 g KH_2_PO_4_, 60 g sucrose, 2 g MgSO_4_·7H_2_O, 400 mg Na_2_HPO_4_·12H_2_O, 40 mg FeSO_4_·7H_2_O, 40 mg CaCl_2_, 0.08 g CuSO_4_·5H_2_O, 0.12 g adenine sulfate, and 4 g agar) was used to determine the strain growth rate and the mycelium branching number.

### Plasmid construction

Plasmids pYSK7, pBD5, and pCRII have been characterized in previous reports (Dörnte and Kües 2016; Dörnte et al. 2020; Rühl et al. 2018). Considering the close evolutionary affinity between *C. cinerea* okayama7#130 and *C. cinerea* FA2222, the genomic information of *C. cinerea* FA2222 is referenced to that of *C. cinerea* okayama7#130 (genome accession number: GCA_000182895.1). The primers used in this study are shown in the Supplementary information file 1: Table S1. The pYSK fragment (the backbone of pYSK-*lcc5*) was obtained by enzymatic digestion of vector pYSK7 with *Bam*H I and *Hpa* I. The *lcc5* fragment was obtained from the *C. cinerea* FA2222 genome using primers *lcc5*-F/*lcc5*-R. Then, fragments pYSK and *lcc5* were transferred into *S. cerevisiae* Y1H to construct recombinant vector pYSK-*lcc5* based on homologous recombination.

Similarly, vectors pYSK-anti*ChiEn1* and pYSK-anti*ChiE2* for silencing *ChiEn1* and *ChiE2* were obtained when fragment *lcc5* was replaced by fragments anti*ChiEn1* and anti*ChiE2*, respectively. In which, fragments anti*ChiEn1* and anti*ChiE2* were obtained by PCR amplification with primer pairs anti*ChiEn1*-F/anti*ChiEn1*-R and anti*ChiE2*-F/anti*ChiE2*-R, respectively, using the cDNA of *C. cinerea* FA2222 as template. Theoretical sequences of fragments anti*ChiEn1* and anti*ChiE2* were generated by the antisense RNA sequence prediction function of an online website (http://rnaidesigner.thermofisher.com/rnaiexpress/). The *ChiEn1* and *ChiE2* RNA interference fragments anti*ChiEn1* and anti*ChiE2* are shown in the Supplementary information file 1: Table S2.

### *C. cinerea* protoplast preparation and transformation

The *C. cinerea* protoplast preparation and transformation were performed according to the method previously reported by Dörnte et al. (2020). Briefly, the protoplasts were prepared based on the enzymatic digestion of *C. cinerea* oidia until the enzymatic extent reached 40–60%. Plasmid DNA for transformation was isolated from *E. coli* JM109 by alkaline lysis. The plasmid was then transferred into the protoplasts mediated by PEG and positive transformants were obtained by protoplast regeneration.

### *C. cinerea* cultivation

For shake flask fermentation, 4 pieces of appropriately sized mycelium blocks (side length of about 1 cm) were inoculated into a 500-mL triangular flask containing 100-mL YMG medium. The inoculated mixture was then cultured at 37 °C, 200 rpm. For 3-L fermenter fermentation, the mycelium was first collected after 4 days of shake flask culture, then homogenized and inoculated at 5% inoculum into a 3-L fermenter (New Brunswick™ BioFlo®/CelliGen®115) containing 1.5 L mKjalke medium, followed by cultivation at 37 °C, 50–250 rpm, pH 5.8, and 40% dissolved oxygen. It is worth mentioning that 1 mM CuSO_4_ was supplemented to the YMG and mKjalke medium during *C. cinerea* culture to enhance laccase activity.

### *C. cinerea* physiological characteristics determination

Suitable-sized mycelial clumps were inoculated on VI medium plates and the inoculation center point was marked on the plate backside. Then, it was incubated at 37 ℃, and mycelial growth diameter was measured daily to obtain mycelial growth rate data. Mycelial branching numbers were determined by inoculating mycelium onto coverslips dripping with VI medium and incubating at 37 °C for 4–5 days, followed by random observation under a microscope. When the mycelium on the plate reaches the Petri dish edge and begins to produce spores, rinse the Petri dish with sterile water. The mycelium and filtrate supernatant were then removed by filtration and centrifugation, respectively. Finally, spore precipitates were resuspended with sterile water and counted using a hemocytometer plate.

Since Congo red forms a complex with the chitin in the fungal cell wall that prevents further aggregation, it hinders mycelial growth when the structural integrity of the cell wall is altered and its sensitivity is increased (Cairns et al. 2023; Ram and Klis 2006). To further investigate whether chitinase gene silencing would affect cell wall integrity, silenced strains were inoculated on PDA plates supplemented with 200 mg/L Congo red, while wild-type *C. cinerea* FA2222 was used as a control.

### Atomic force microscopy (AFM) analysis

Sample preparation methods for AFM (NanoScope VIII, ICON) assay as reported by Zhao et al. (2005). Wherein, the mycelium was scanned in touch to locate the position for indentation experiments, and force profiles were collected at 0.5 µm/s *z* scan rate, 250 nm *z* scan size, and approximately 40 nm cantilever beam degree. AFM image analysis performed by NanoScope Analysis software (v1.8, Bruke Co., Billerica, MA, USA). Since cells are not homogeneous objects, there are certain differences in the viscoelastic properties of individual sub-partitions. Therefore, two diagonals along the sample dimensions were first set as X- and Y-axes, respectively. The instantaneous Young’s modulus of the sample was then counted at all measurement points on the X- and Y-axes to plot the distribution histograms.

### *C. cinerea* biomass determination

A defined volume of the fermentation mixture was taken from the fermentation system, filtered to remove the liquid, and then dried to equilibrium weight at 90 °C. The ratio between the final weight and the sampling volume is the *C. cinerea* biomass (g/L).

### Cell wall chitin content determination

Mycelium from liquid cultures was collected, ground to powder with liquid nitrogen, and resuspended in water for ultrasonic crusher crushing. Take 0.5 g of the above mixture freeze-dried powder and treat it with 6 M hydrochloric acid for 1 h at room temperature. Then, the remaining hydrochloric acid was removed by a rotary evaporator and 2 mL of sterile water was added. After centrifugation, 1-mL supernatant was added to 0.25-mL acetylacetone solution at 4% volume fraction, and a water bath at 90 ℃ for 1 h. After cooling to room temperature, 2-mL hexanol and 0.25-mL Ehrlich reagent were added, and the absorbance values of the obtained mixture were measured at 530 nm. Finally, the cell wall chitin content, i.e., the amount of glucosamine hydrochloride (GAH) per gram of biomass (mg/g), was calculated from the GAH standard curve.

### Laccase activity determination

The laccase activity assay method used in this study was modified accordingly from the previous report (Hu et al. 2019). Briefly, 17 μL fermentation supernatant was mixed with 33 μL of 15 mM 2,2′-azino-bis (3-ethylbenzothazoline-6-sulfonate) (ABTS) and 950 μL sodium tartrate. The mixture was then reacted in a water bath at 30 °C for 3 min and immediately cooled in ice water. Its absorbance value was measured at 420 nm. One activity unit (U) was defined as the amount of laccase required for oxidizing 1 μmol of ABTS per minute. The formula for calculating laccase activity based on the above method is OD_*420*_ × dilution factor × 555.56 (U/L). In addition, laccase was purified according to the method of Chen et al. (2016) to obtain pure enzymes for specific activity determination.

### Native-PAGE and SDS-PAGE analysis

Native-PAGE and SDS-PAGE assays for laccase were performed according to our previously reported methods (Liu et al. 2022; Xu et al. 2019a). After electrophoresis, the active gel was immersed in 100-mM citrate–phosphate buffer containing 15-mM ABTS, and incubated at 30 ℃ until a green band appeared, and the denatured gel was stained with Coomassie Brilliant Blue R-250.

### Glucose determination

Glucose concentrations in culture supernatants were determined directly with automatic biosensors (Sieman Technology Co., Ltd, Shenzhen, China) following the manufacturer’s instructions.

### Quantitative reverse transcription-PCR (qRT-PCR) analysis

*C. cinerea* mycelia in the fermentation broth samples were collected by filtration, and then their total RNA and corresponding cDNA were obtained using RNAiso-Plus extraction reagent (TaKaRa, Dalian, China) and Evo M-MLV RT kit (AG, Hunan, China), respectively. The qualities of the cDNA samples were measured using a Qubit 4.0 fluorometer and Qubit dsDNA HS Assay Kit (Invitrogen, USA). To normalize the qRT-PCR data, the *β-actin* gene was chosen as the reference based on our previous study (Liu et al. 2022). Primers used for qPCR amplification of the chitinase and β-actin genes are shown in the Supplementary information file 1: Table S1. qPCR was performed based on the Light-Cycler 96 Real-Time PCR system (Roche, Basel, Switzerland) using the SYBR Green Premix Pro Taq HS qPCR Kit (AG, Hunan, China). The qPCR amplification conditions included 95 ℃ for 30 s, followed by 35 cycles of 95 ℃ for 5 s, 60 ℃ for 30 s, and a melt-curve step (0.3 ℃/s, from 60 to 95 ℃). Real-time PCR data were calculated using a 2^−ΔΔCT^ methodology (Livak and Schmittgen 2001). The transcript levels of each gene are presented separately as their fold change relative to that of the internal reference gene *β-actin*.

### Statistical analysis

All data in this study are from three independent experiments and are presented as their mean (± standard deviation). Data were statistically analyzed using SAS statistical software (v8.1, SAS Institute Inc., Cary, NC, USA) based on the Student *t*-test, where differences of *p* < 0.05 were considered statistically significant.

## Results

### Recombinant production of Lcc5 in *C. cinerea*

**Fig. 1:**
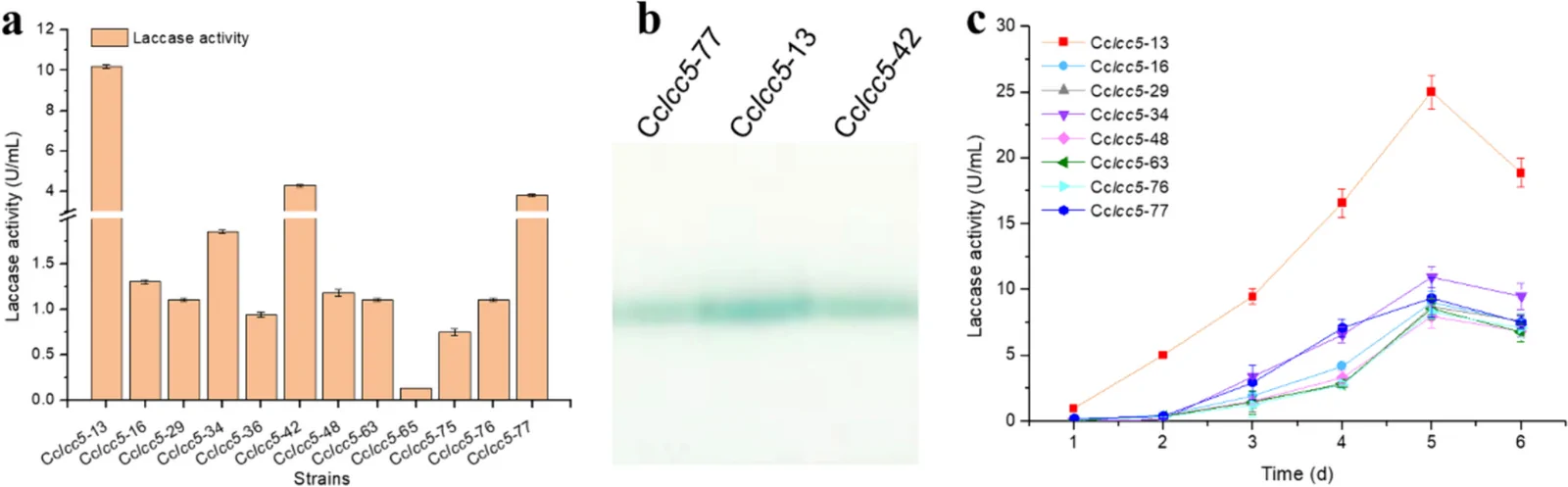
*C. cinerea* recombinant strains shake flask fermentation for Lcc5 production. (**a**) Extracellular Lcc5 activity of recombinant strains based on YMG medium. (**b**) Native-PAGE analysis results of fermentation supernatant. (**c**) Lcc5 production profile of recombinant strains based on mKjalke medium. Error bars represent the standard deviation

The production strain Cc*lcc5* was constructed by co-transforming the vectors pYSK-*lcc5* and pBD5 into *C. cinerea* FA2222, and a total of 12 positive transformants were obtained (Fig. 1a). As shown in Fig. 1a, after 4 days of shake-flask culture based on YMG medium, the highest extracellular laccase activity was produced by Cc*lcc5*-13 (10.2 U/mL), followed by Cc*lcc*5-42 (4.3 U/mL), and Cc*lcc5*-77 (3.8 U/mL). The extracellular laccase activity of Cc*lcc5*-13 is 78.5-fold higher than that of Cc*lcc5*-65 (0.13 U/mL), which had the lowest extracellular activity. Then, the fermentation supernatants of the three strains (Cc*lcc5*-13, Cc*lcc5*-42, and Cc*lcc5*-77, respectively) with the highest extracellular laccase activity were selected for native-PAGE assay. The native-PAGE results of these strains were consistent with their extracellular laccase activity (Fig. 1b). The theoretical protein molecular weight of laccase Lcc5 is approximately 57.5 kDa. Since there is no clear correspondence between the position of the protein bands on native-PAGE and its molecular weight, the protein molecular weight marker was not labeled.

To investigate the effect of medium components on the recombinant Lcc5 production, eight recombinant strains (Cc*lcc5*-13, Cc*lcc5*-16, Cc*lcc5*-29, Cc*lcc5*-34, Cc*lcc5*-48, Cc*lcc5*-63, Cc*lcc5*-76, and Cc*lcc5*-77, respectively) with high laccase activity were selected for shake flask fermentation based on mKjalke medium. As shown in Fig. 1c, the extracellular laccase activity of different strains ranged from 8 to 25 U/mL. Among them, the extracellular laccase activity of Cc*lcc5*-13 was significantly higher than that of other strains, reaching a maximum value of 25 U/mL. Therefore, follow-up studies were performed based on Cc*lcc5*-13 only.

### Identification of the critical chitinase genes

**Fig. 2:**
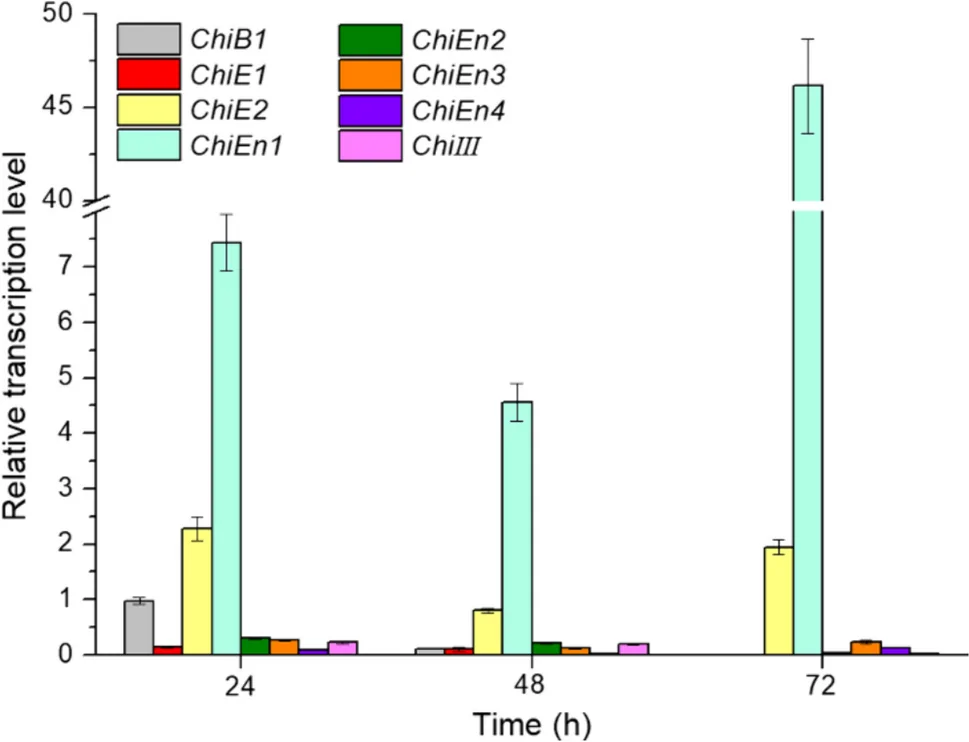
Chitinase transcript levels in the *C. cinerea* FA2222. Error bars represent the standard deviation

The transcriptional levels of eight chitinase genes (*ChiB1*, *ChiE1*, *ChiE2*, *ChiEn1*, *ChiEn2*, *ChiEn3*, *ChiEn4*, and *ChiIII*, respectively) in the genome of *C. cinerea* FA2222 were measured at different culture times during shake flask fermentation. The accession numbers of the chitinase genes referenced in this study are shown in the Supplementary information file 1: Table S3. As shown in Fig. 2, the transcription levels of *ChiE2* and *ChiEn1* were significantly up-regulated, reaching a maximum at 24 and 72 h, respectively. Furthermore, the transcription levels of *ChiE2* and *ChiEn1* were tens to hundreds of times higher than those of other chitinase genes, suggesting that ChiEn1 and ChiE2 may play a major role in the *C. cinerea* cell wall remodeling.

### Construction and characterization of chitinase-silenced strains

The vectors pYSK-anti*ChiEn1* and pYSK-anti*ChiE2*, which silenced the *ChiEn1* and *ChiE2* expression, were co-transformed into *C. cinerea* FA2222 with pBD5, respectively, to construct chitinase-silenced engineered strains Ccanti*ChiEn1* and Ccanti*ChiE2*. Ultimately, 21 and 14 positive transformants were obtained, respectively. To confirm the interference effectiveness, we randomly selected one-fourth of the above positive transformants (i.e., there are 5 strains of Ccanti*ChiEn1* and 4 strains of Ccanti*ChiE2*, respectively) for subsequent validation.

**Fig. 3:**
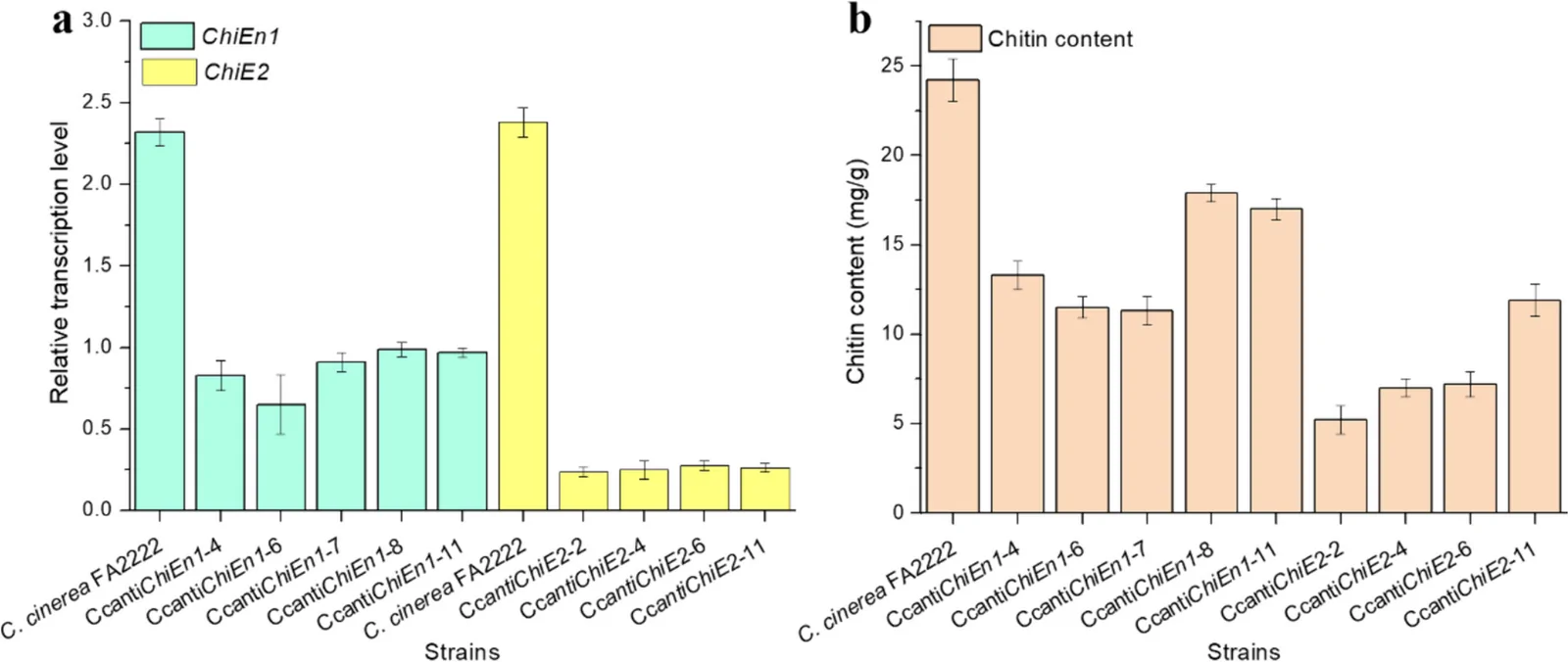
Chitinase gene transcript levels and chitin content in RNAi strains. (**a**) *ChiEn1* and *ChiE2* transcript levels. (**b**) Cell wall chitin content. Error bars represent the standard deviation

Mycelial samples of wild-type and chitinase-silenced strains were collected after a 60-h shake flask incubation to determine chitinase gene (*ChiEn1* or *ChiE2*) transcript levels and chitin content. The qRT-PCR results showed that *ChiEn1* and *ChiE2* transcript levels in Ccanti*ChiEn1* and Ccanti*ChiE2* strains were reduced by about 45% and 80%, respectively, compared to *C. cinerea* FA2222 (Fig. 3a). In addition, Ccanti*ChiEn1* and Ccanti*ChiE2* had significantly lower chitin content than *C. cinerea* FA2222 (5–20 mg/g vs 25 mg/g, Fig. 3b). These results show that Ccanti*ChiEn1* and Ccanti*ChiE2* exhibit good chitinase silencing and also demonstrate that *ChiEn1* and *ChiE2* are indeed the critical chitinase genes affecting the chitin content of *C. cinerea* FA2222 cell wall.

**Fig. 4:**
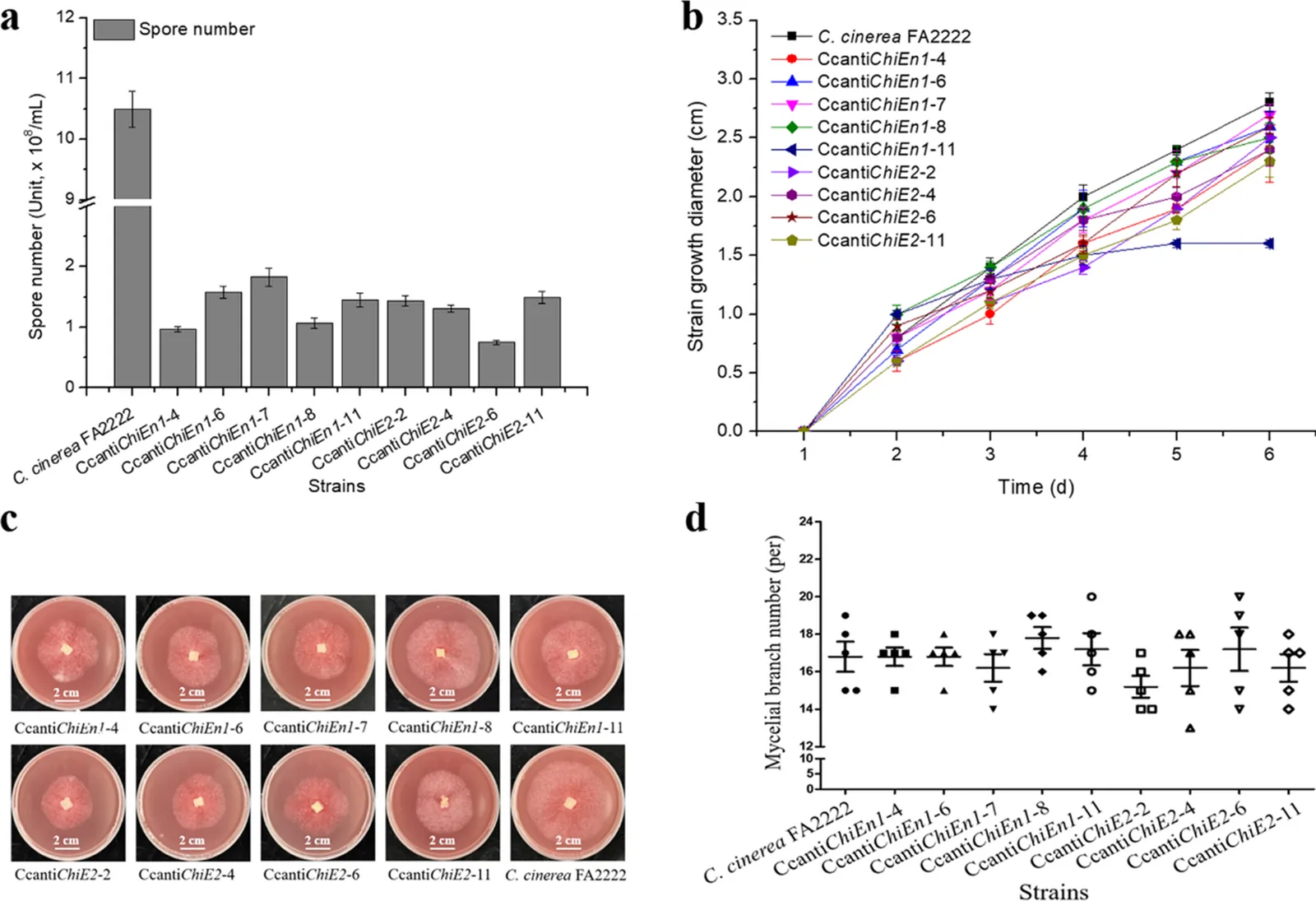
Mycelial physiology and morphology of chitinase gene silenced strains. (**a**) Sporulation capacity. (**b**) Growth rate. (**c**) Cell wall sensitivity. (**d**) Mycelial branching number. Error bars represent the standard deviation

Then, the effects of chitinase gene silencing on the mycelial physiological properties were investigated in terms of spore yield, growth rate, branch number, and cell wall sensitivity, respectively (Fig. 4). The results showed that compared to *C. cinerea* FA2222, both Ccanti*ChiEn1* and Ccanti*ChiE2* decreased spore formation and growth rate (Fig. 4a and b), and increased cell wall sensitivity (Fig. 4c), but no significant difference in mycelial branching (Fig. 4d).

### Effect of chitinase silencing on Lcc5 recombinant production

**Fig. 5:**
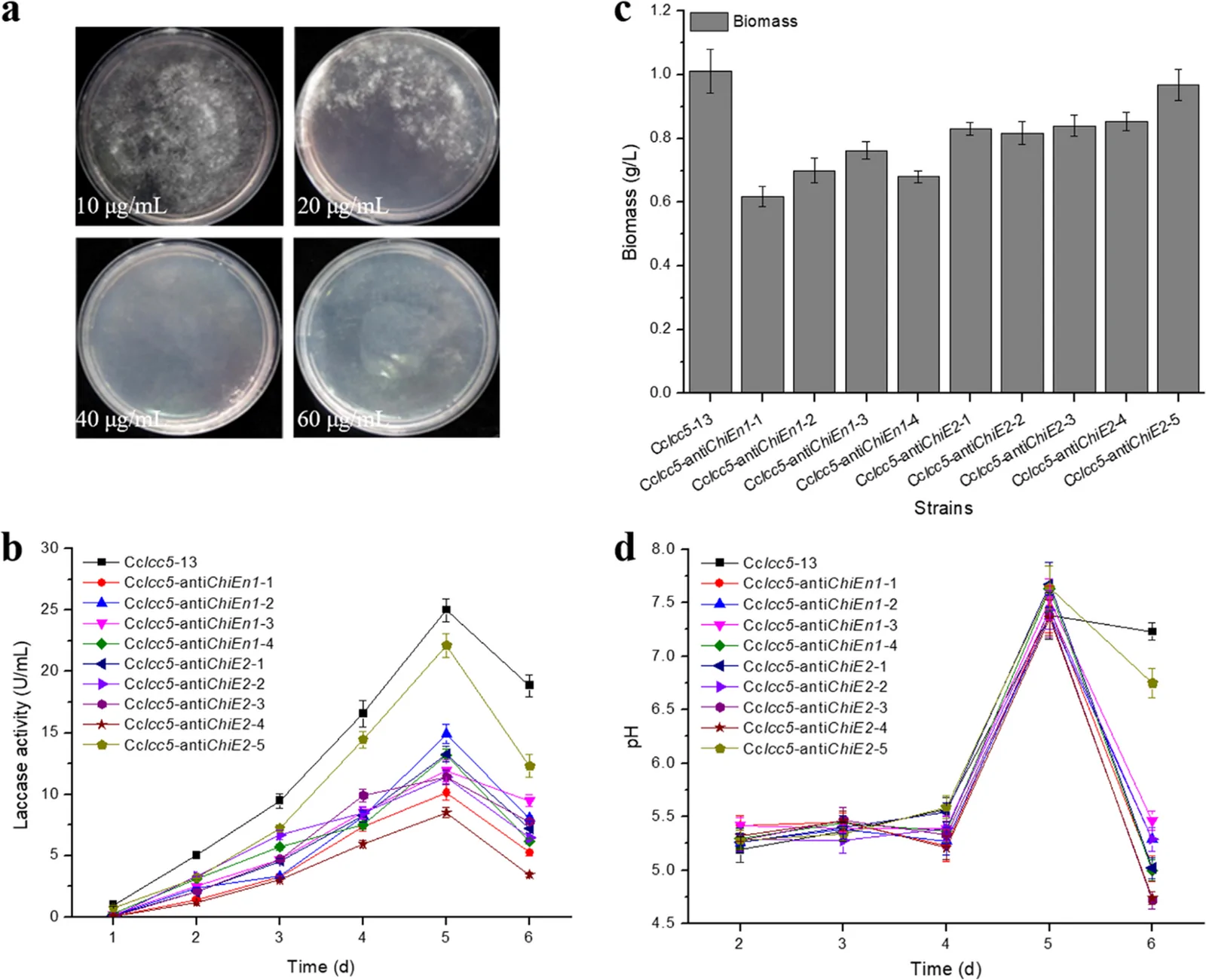
Shake flask fermentation of chitinase gene silenced recombinant strains. (**a**) Hygromycin minimum effective concentration. (**b**) Extracellular laccase activity profile. (**c**) Biomass. (**d**) Fermentation pH profile. Error bars represent the standard deviation

Recombinant Lcc5 production strains, Cc*lcc5*-anti*ChiEn1* or Cc*lcc5*-anti*ChiE2*, were constructed by co-transforming interference vectors pYSK-anti*ChiEn1* or pYSK-anti *ChiE2* with pCRII containing the hygromycin resistance gene into Cc*lcc5*-13. To determine the minimum effective amount of hygromycin in the screening plates, we first investigated the hygromycin tolerance of Cc*lcc5*-13. Cultivation of Cc*lcc5*-13 on screening plates containing different hygromycin additions (10, 20, 40, and 60 μg/mL, respectively) revealed that the lowest inhibitory concentration of hygromycin against Cc*lcc5*-13 was 40 μg/mL (Fig. 5a).

After screening and validation, Cc*lcc5*-anti*ChiEn1* and Cc*lcc5*-anti*ChiE2* finally obtained 4 and 5 positive transformants, respectively. After shake flask culture based on mKjalke medium, the extracellular laccase activities of Cc*lcc5*-anti*ChiEn1* and Cc*lcc5*-anti*ChiE2* were in the range of 10–15 and 8–22 U/mL, respectively (Fig. 5b). The extracellular laccase activity of Cc*lcc5*-anti*ChiE2*-5 was the highest at 22 U/mL, but still lower than that of Cc*lcc5*-13 (25 U/mL, Fig. 5b). The results were further confirmed by SDS–PAGE analysis (Supplementary information file 1: Fig. S1). The Lcc5 produced by Cc*lcc5*-13 and Cc*lcc5*-anti*ChiE2* have similar specific activities, 22.4 and 21.5 U/μg, respectively. At shake flask fermentation for 60 h, the transcript level of *ChiE2* in Cc*lcc5*-anti*ChiE2*-5 was lower than that in Cc*lcc5*-13 (Supplementary information file 1: Fig. S2).

In addition, at the end of shake flask fermentation, both the biomass and pH of Cc*lcc5*-anti*ChiEn1* and Cc*lcc5*-anti*ChiE2* were lower than those of Cc*lcc5*-13 (Fig. 5c and d). Among all the chitinase-silenced strains, only Cc*lcc5*-anti*ChiE2*-5 showed biomass and pH closest to those of Cc*lcc5*-13 (Fig. 5c and d).

### Effect of chitinase silencing on Lcc5 recombinant production strain morphology

**Fig. 6:**
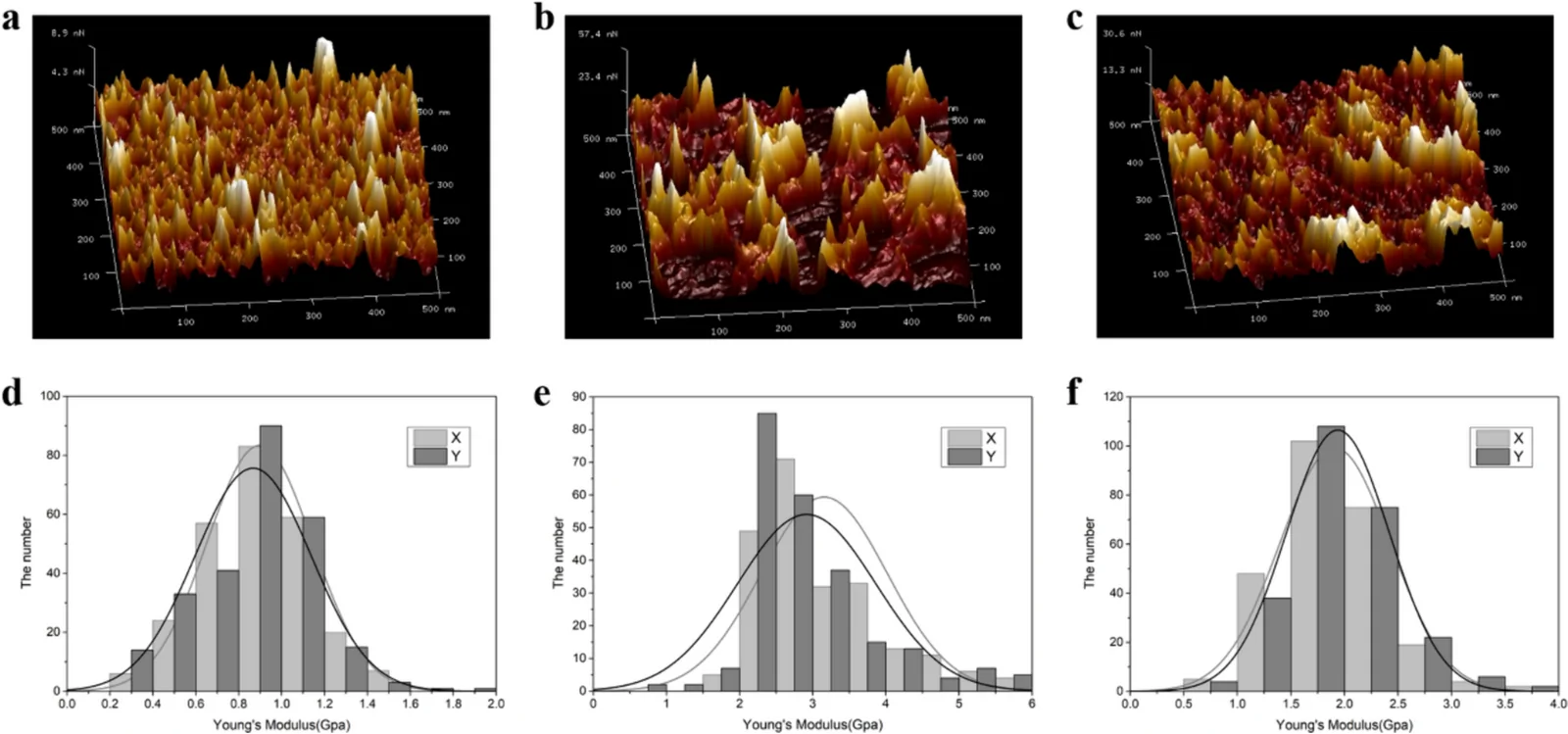
Roughness and shear tolerance of mycelial cell wall. (**a**), (**b**), and (**c**) are the roughness 3D graphs for Cc*lcc5*-13, Cc*lcc5*-anti*ChiEn1*-2, and Cc*lcc5*-anti*ChiE2*-5, respectively. (d), (e) and (f) are Young's modulus information for Cc*lcc5*-13, Cc*lcc5*-anti*ChiEn1*-2, and Cc*lcc5*-anti*ChiE2*-5, respectively. Standard deviations in the analyses were always below 20% (not further shown)

In this section, only Cc*lcc5*-anti*ChiEn1*-2 and Cc*lcc5*-anti*ChiE2*-5 with the highest extracellular laccase activity were selected as research objects (Fig. 5b). Their mycelial morphology and tolerance to external shear forces were examined separately using AFM. The results showed significant differences in cell wall roughness between the silent and wild strains (Fig. 6a, b, and c). Among them, the arithmetic roughness (R_a_), root mean square roughness (R_q_), and maximum roughness (R_max_) of Cc*lcc5*-13, Cc*lcc5*-anti*ChiEn1*-2, and Cc*lcc5*-anti*ChiE2*-5 are shown in Table 1.

**Table 1:** Roughness parameter information

Recombinant	R_a_/nN	R_q_/nN	R_max_/nN
Cc*lcc5*-13	0.93	1.24	11.40
Cc*lcc5*-anti*ChiEn1*-2	8.23	10.50	69.2
Cc*lcc5*-anti*ChiE2*-5	4.04	5.10	35.6

In addition, Young’s modulus information showed that the tolerance of Cc*lcc5*-13 was more concentrated at 0.8–1.2 Gpa, whereas Cc*lcc5*-anti*ChiEn1*-2 and Cc*lcc5*-anti*ChiE2*-5 were significantly more tolerant, mainly around 2–3 Gpa (Fig. 6d, e, and f). These suggest that the cell walls of silenced strains can withstand greater shear forces, and then a higher agitation speed may be beneficial to increase their extracellular laccase production. Therefore, we will use a 3-L fermenter to explore the Lcc5 production levels of silenced and wild strains at different agitation speeds. Since Cc*lcc5*-anti*ChiE2*-5 showed the highest extracellular laccase activity, it was selected only for the follow-up study.

### Effect of 3-L fermenter agitation speed on *Lcc5* recombinant production

**Fig. 7:**
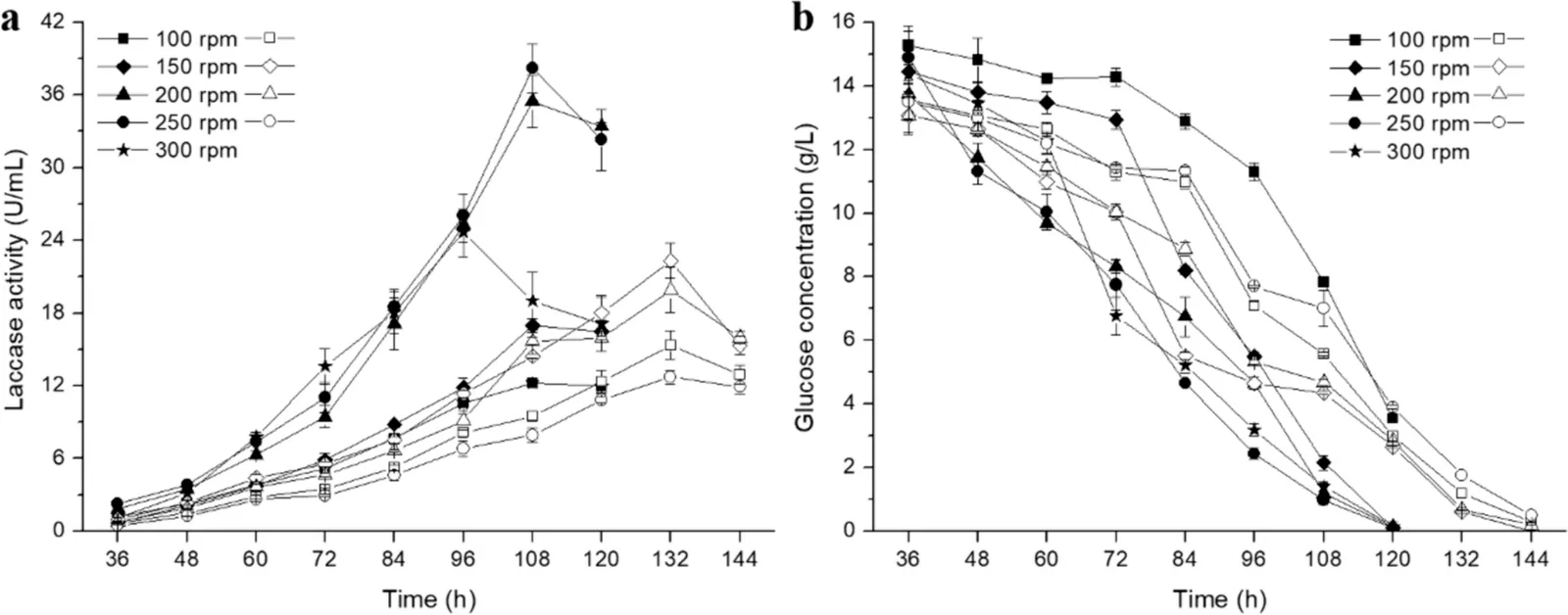
Scale-up (3-L) fermentation of *C. cinerea* recombinant strains. (**a**) Extracellular laccase activity profile. (**b**) Glucose concentration profile. Solid for Cc*lcc5*-anti*ChiE2*-31, open for Cc*lcc5*-13. Error bars represent the standard deviation

Cc*lcc5*-anti*ChiE2*-5 and Cc*lcc5*-13 were cultured in 3-L fermenters at different agitation speeds, respectively (Fig. 7). The extracellular laccase activities of Cc*lcc5*-13 and Cc*lcc5*-anti*ChiE2*-5 reached a maximum of 25.5 and 38.2 U/mL at 150 and 250 rpm, respectively, corresponding to productivity of 0.19 and 0.35 U/mL·h (Fig. 7a). Extracellular laccase activity and productivity of Cc*lcc5*-anti*ChiE2*-5 are 1.5- and 1.8-fold greater than those of Cc*lcc5*-13, respectively. Remarkably, the extracellular laccase activity of Cc*lcc5*-anti*ChiE2*-5 was 2.6-fold higher than that of Cc*lcc5*-13 (14.5 U/mL) when both agitation speeds were 250 rpm (Fig. 7a). Furthermore, as shown in Fig. 7b, a negative correlation between laccase activity and glucose residue was observed in recombinant strain fermentation supernatant.

## Discussion

Due to its unique properties, *C. cinerea* is widely used for producing enzymes related to lignin degradation, such as laccase. In this study, the laccase Lcc5, which is valuable for feed detoxification and wine lees feed processing, was homologous recombinantly expressed in *C. cinerea* FA2222. In addition, the effects of critical chitinase genes in the *C. cinerea* FA2222 genome on its morphology and Lcc5 recombinant production were investigated using RNAi.

As shown in Fig. 1, there were significant differences in the extracellular laccase activities of the various *C. cinerea* transformants. Since the recombinant expression vector pYSK-*lcc5* is constructed based on pYSK7 (Kilaru et al. 2006), which does not contain site-specific homologous arm sequences, and the expression host *C. cinerea* FA2222 is not Ku protein deficient (Tu et al. 2021), homologous recombination is not easily achieved. Furthermore, as shown in Fig. 3a, even when the same chitinase silencing vectors were transformed into the same host strain, there were significant differences in the chitinase transcript levels of the different transformants obtained. Therefore, it is reasonable to speculate that the random integration of the pYSK-*lcc5* expression vector into the *C. cinerea* FA2222 genome may be responsible for the significant differences in extracellular laccase activity among different *C. cinerea* transformants.

In addition, *C. cinerea* transformants had higher extracellular laccase activity in the mKjalke medium (Fig. 1a) compared to the YMG medium (Fig. 1c), which might be attributed to the richer total carbon and nitrogen (C + N) sources as well as higher carbon to nitrogen (C/N) ratio of mKjarke medium. Similar results were reported by Rühl et al. (2013), who also found that the mKjalke medium was more suitable for the laccase Lcc1 and Lcc5 production by *C. cinerea* FA2222 compared to the YMG medium. Furthermore, they thought that the C/N ratio might be more important than the C + N sources in regulating laccase expression (Rühl et al. 2013).

*ChiEn1* and *ChiE2*, the two chitinase genes with the highest transcriptional activity in the *C. cinerea* FA2222 genome, were used as subjects (Fig. 2). Although the transcriptional activity of *ChiEn1* was higher than that of *ChiE2* in *C. cinerea* FA2222 (Fig. 2), the average chitin content in the cell wall of Ccanti*ChiE2* silenced with *ChiE2* was lower than that of Ccanti*ChiEn1* silenced with *ChiEn1* (Fig. 3b). This may indicate that *ChiE2* has a greater role in controlling cell wall chitin content.

Previous studies have shown that filamentous fungal mycelia elongate by inserting new chitin microfilaments into the original structure, a process that requires the continuous unfastening and resealing of the chitin-glucan linkage catalyzed by relevant enzymes such as chitinase (Kües 2000). In addition, conidial production in filamentous fungi involve cell wall structural remodeling. Therefore, repressing chitinase activity may lead to a decrease in cell wall chitin content, mycelial growth rate, and conidial number. The results of the present study are consistent with the above speculations, i.e., compared to *C. cinerea* FA2222, both Ccanti*ChiEn1* and Ccanti*ChiE2* had lower cell wall chitin content (Fig. 3b), conidial numbers (Fig. 4a) and mycelial growth rates (Fig. 4b) but increased cell wall sensitivity (Fig. 4c). A similar phenomenon was reported previously by Jaques et al. (2003), they found that knocking out the chitinase ChiA resulted a reduction in conidial and mycelial growth of *Aspergillus nidulans*.

With increasing *C. cinerea* extracellular laccase activity in this study, the medium pH was first acidic and then gradually became alkaline, and the extracellular laccase activity reached its maximum value when the medium pH was most alkaline (pH about 7.7, Fig. 5b and d). Combined with the report of Rühl et al. (2018), it is not difficult to speculate that this might be caused by high glucose consumption and reduced C/N ratio in the medium. Furthermore, as shown in Fig. 4c, chitinase silencing caused an increase in mycelial cell wall sensitivity, which would reduce the viability of the chitinase-silenced recombinant strains during shake-flask fermentation, leading to a decrease in biomass (Fig. 5b) and extracellular laccase activity (Fig. 5c). However, variations in the vitality and biomass of recombinant strains resulted in their different nutrient consumption from the fermentation medium, resulting in different fermentation pH. In summary, chitinase may affect *C. cinerea* extracellular laccase activity and fermentation pH by influencing the vitality and biomass of recombinant strains. As for the reason why different chitinase-silenced recombinant strains exhibit distinct fermentation characteristics (Fig. 5b, c and d), it may be due to the random integration of the chitinase silencing vector into the Cc*lcc5*-13 genome.

R_a_, R_q_, and R_max_ are common parameters for characterizing roughness (Moura et al. 2021). Therein, R_a_ is the absolute value of the height deviation measured relative to the center plane in the examined area; R_q_ is the root mean square parameter corresponding to R_a_; R_max_ is the height difference between the highest and lowest points relative to the center line within the contour length in the cross-sectional contour profile graphs. 3D roughness maps of mycelial cell walls showed that the lower the extracellular laccase activity, the higher the roughness of the mycelial pellet surface (Table 1). This is consistent with the study by Rühl et al. (2018). Their study revealed that the extracellular recombinant laccase Lcc1 activity of *C. cinerea* was increased up to 3.2-fold and the mycelial pellet morphology had a smoother surface after shake flask fermentation at 25 °C compared to 37 °C.

Based on Young’s modulus distribution histogram (Fig. 6d, e, and f), it can be seen that silencing chitinase genes increased the shear tolerance of mycelium. Thus, Cc*lcc5*-anti*ChiE2*-5 had higher extracellular laccase activity (Fig. 7a) and consumed more glucose (Fig. 7b) than Cc*lcc5*-13 probably because its cell wall was more shear-resistant and therefore had a higher survival probability at high agitation speed. Moreover, an appropriately high agitation speed also increases the fermentation solution mixing and hence further enhances the extracellular laccase activity of recombinant strains. On the contrary, when the agitation speed was low, the increased sensitivity of mycelium’s cell walls after chitinase silencing (Fig. 4c) might led to a decrease in bioactivity biomass of Cc*lcc5*-anti*ChiE2*-31, which in turn caused a reduction of its glucose consumption and extracellular laccase activity. Similarly, it is possible that the combined effect of mycelial shear tolerance strength and cell wall sensitivity led to an increase in extracellular laccase activity in chitinase-silenced strains that was only observed in scaled-up (3-L) fermentation (Fig. 7), but not in shake-flask fermentations (Fig. 6). This may also explain the reduced biomass (Fig. 5b) and extracellular laccase activity (Fig. 5c) of the chitinase-silenced recombinant strains at the shake flask fermentation level.

Mycelial morphology is an important factor influencing filamentous fungi to produce target product yields under submerged fermentation conditions (Li et al. 2022a). In recent years, cell wall chitin content has emerged as an important target for modulating filamentous fungal mycelium morphology. In this study, high-level production of Lcc5 in *C. cinerea* was achieved by silencing the chitinase gene. By silencing the *ChiE2*, the engineered strain’s growth characteristics were altered and its tolerance to external shear force was increased. The extracellular laccase activity and productivity of the final recombinant strain Cc*lcc5*-anti*ChiE2*-5 obtained in a 3-L fermenter culture were 1.6-fold and 1.8-fold higher than those of the control Cc*lcc5*-13, respectively. The high-producing strain constructed here will pave the way for scale-up production of laccase Lcc5. Moreover, this study also provides a novel strategy for enhancing other target product yields in *C. cinerea*.

## Supplementary information

Below is the link to the electronic supplementary material.

Supplementary file1 (PDF 189 KB)

## Data Availability

All data generated or analyzed during this study are included in this published article and its additional files: Supplementary material.
