# Supplementary material for: Enhanced extracellular production of laccase in Coprinopsis cinerea by silencing chitinase gene
Source: Appl Microbiol Biotechnol. 2024 May 7;108(1):324. doi: 10.1007/s00253-024-13164-9 (PMC11076350; doi:10.1007/s00253-024-13164-9)
Supplement: Supplementary file 1 — Supplementary file1 (PDF 189 KB) [file 253_2024_13164_MOESM1_ESM.pdf]

Additional information

Enhanced extracellular production of laccase in *Coprinopsis cinerea* by silencing chitinase gene

Dongbang Yao[1,2,3], Yuting Ma[1,2], Jie Ran[1,2], Jiaxiu Wang[1,2], Ursula Kües[4], Juanjuan Liu[1,2,3], Danya Zhou[5], Xuecheng Zhang[1,2,3], Zemin Fang[1,2,3*], Yazhong Xiao[1,2,3*]

*Correspondence: Zemin Fang (zemin_fang@ahu.edu.cn), Yazhong Xiao (yzxiao@ahu.edu.cn)

[1] School of Life Sciences, Anhui University, Hefei, 230601, China.

[2] Anhui Key Laboratory of Modern Biomanufacturing, Hefei, 230601, China.

[3] AHU Green Industry Innovation Research Institute, Hefei, 230088, China.

[4] Molecular Wood Biotechnology and Technical Mycology, Büsgen-Institute and Goettingen Center for Molecular Biosciences, University of Goettingen, Büsgenweg 2, 37077 Goettingen, Germany.

[5] School of Basic Medical Sciences, Zhengzhou University, Zhengzhou, 450001, China.

SUPPORTING INFORMATION

Table S1 Primers used in this study

Primer	Sequence (5'-3')
lcc5-F	CTCCCATCTACACACAACAAGCTTATCGCCAT GTCGTTTGCTTGGAAAGCATTGGC
lcc5-R	CACTGGCCCTCTGGTCAACTATAATATTATTTA GGGATACATAGGGAGCAAGTTCGAA
anti*ChiEn1*-F	CTCCCATCTACACACAACAAGCTTATCGC CTGAGCTTCCCTCCCGTAGGATCTTG
anti*ChiEn1*-R	CACTGGCCCTCTGGTCAACTATAATATTAT AGTACCAGCGGAACCTGACTCGGA
anti*ChiE2*-F	CTCCCATCTACACACAACAAGCTTATCGC CTCGGCAGTGGGTACGGCAAACG
anti*ChiE2*-R	CACTGGCCCTCTGGTCAACTATAATATTAT TCTATGGTTATTTCTCTCCTTTGCGCTG
qRT-*β-actin*-F	CTCTGGAGTTATGGTAGGAATGGGC
qRT-*β-actin*-R	GATGCCATGTTCGATGGGGTACTTG
qRT-*ChiE1*-F	AGAGGGAGAACGGCGAAGGATGCAA
qRT-*ChiE1*-R	CAGGTTGGCTTGGTGGTTCGCAGT
qRT-*ChiE2*-F	TCTTCACCGTTCGCTTCTGTACTGG
qRT-*ChiE2*-R	TAACTAGAAAGAGCGCTGGCTTCCG
qRT-*ChiIII*-F	TTGGGTGGTCGATCGGATATGAGGC
qRT-*ChiIII*-R	GACTTGGTTGAGGACCTCTCCCAG
qRT-*ChiB1*-F	ACCGGCAAAGCCAACTTCGCCTACT
qRT-*ChiEn1*-F	CCTGGGACAAGTATAATGCCATGACGTT
qRT-*ChiEn1*-R	AGATCCAGTCCATCCACCAAGCGAA
qRT-*ChiEn2*-F	AGATCCCTTGGGAGAAGTACACCGA

Table S1 (continued)

Primer	Sequence (5'-3')
qRT-*ChiEn2*-R	AACGGCCGGAGAGAAGTATTGGGAA
qRT-*ChiEn3*-F	AGGGTAAGCTGGAAGAAGTACACGC
qRT-*ChiEn3*-R	AGTCCATCCACCAATCGAAATCAAGGC
qRT-*ChiEn4*-F	TGGTACACTGGCTGGCATTCGAGA
qRT-*ChiEn4*-R	CAACGTCGAGGAGGGTGTTCACAAA

Table S2 Anti*ChiEn1* and anti*ChiE2* fragment sequence information

**Fragment**	**Sequence (5'-3')**
anti*ChiEn1*	TGAGCTTCCCTCCCGTAGGATCTTGCCTCAGCTCTTGCAAAAACGCAA<br>GGAAGTTGGCGGAATCGGCGTTGGAGATGTGGTTACACCCAATGCCTT<br>GTTTATTGGGAAATTCCCAGTCAAAGTCGATACCATCAAGGTTGTACTG<br>CGTAGCCAAATCCACGACGGCCTTGACGAACGCTGTTCGTCGCTCAGG<br>AGTCGAAACGTGGTCGGAGAAGTAGATAGATCCAGTCCATCCACCAAG<br>CGAAAGTAGGGCTTTCACATTATGCTGCTTAGCCTGTTCCACAAACTTG<br>GGCAACAAAGCCTGACTCTCAGCGTCAAGAGCCAACGGATTAGCCGG<br>ATCCGAAGTCGTCGTCGCAAAAGCAAACGTCATGGCATTATACTTGTC<br>CCAGGACAAACTCTCGGGAGGGTGGGCAGCGGCCTGCCAACTGGGGT<br>ACCAAGCAGTGGCAACCTTTCCCCCAGCATCCAACGATGCCAGGACTT<br>GCGTGGCGTTCAAGCGCACCTCCGAGTCAGGTTCCGCTGGTACT
anti*ChiE2*	TCGGCAGTGGGTACGGCAAACGCAAAATCGATCCAGTCGTATCTCCCG<br>TAGTCCAGTGATTCAGGGGGGAAGGTGTCCGCTGCCCAATCTGGGTAG<br>TAACCCATGAATAGAGGCAGTGGGTTGCTCGGAGTATCCGTTGCCTGA<br>GCACCAGATCCAGTGATGCTGTCAGATCTTTGAGTGCCGCTGCCAGGA<br>TGAGGAAGAATCTTCAATGAATCTCCCTGAATGACTACTGGCAGATCG<br>GGACGTCCAGGCGCGGTGCTACGCTGATGAGCTGCAGTAAACGTAACT<br>GGTGATAAGGACGTCGACGTCGGTGAATCTAAGCCAGAAGTCGATATG<br>ATGCTTGGAGCTGGAGACGACGACAAAGGATGAAAAGGAGGTTCGAT<br>ACGGAAGGGTATCTC

Table S3 Reference gene accession number for the chitinase gene

Gene	Accession number
ChiB1	EAU86796.2
ChiE1	EAU80760.2
ChiE2	EAU84887.2
ChiEn1	EAU81461.1
ChiEn2	EAU81455.1
ChiEn3	EAU84319.1
ChiEn4	EAU91084.2
ChiIII	EAU93428.2

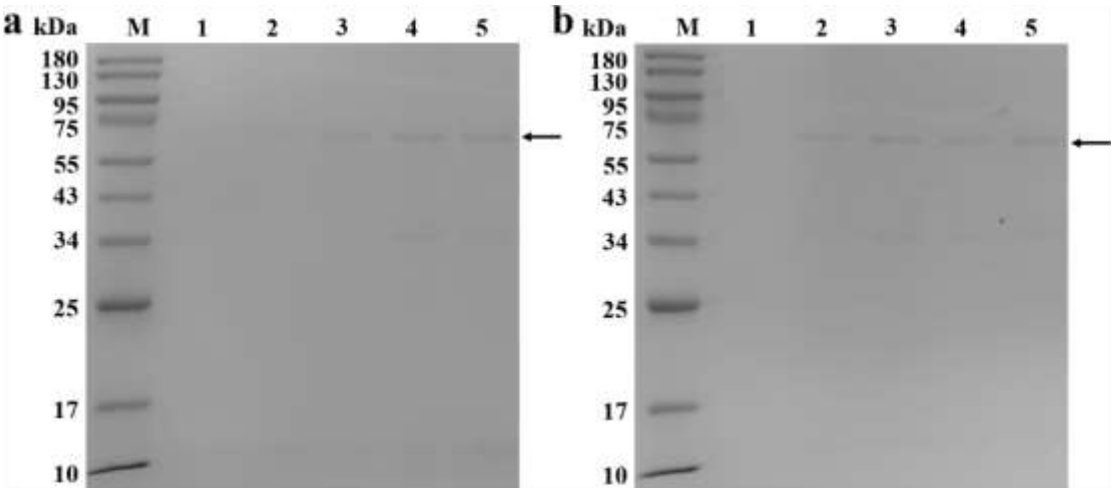

Fig. S1 SDS-PAGE analysis of *C. cinerea* fermentation supernatants

(a) SDS-PAGE analysis of Cc*lcc5*-13 fermentation supernatants. (b) SDS-PAGE analysis of Cc*lcc5*-anti*ChiE2* fermentation supernatants. Lane M: protein molecular weight standard. Lanes 1-5: supernatant samples at 1, 2, 3, 4, and 5 d, respectively. The arrow indicates the band corresponding to Lcc5 (~ 57.5 kDa).

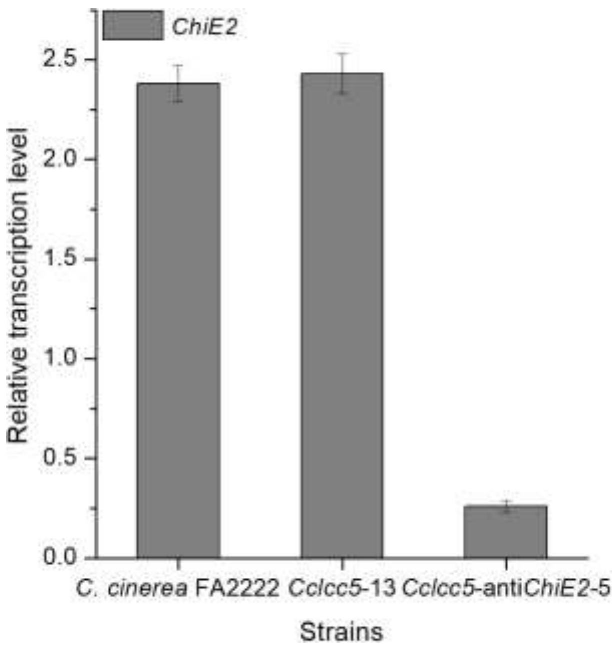

Fig. S2 Chitinase gene transcript level in *C. cinerea*

Error bars represent the standard deviation.
